# Physiological and Proteomics Analyses Reveal the Mechanism of *Eichhornia crassipes* Tolerance to High-Concentration Cadmium Stress Compared with *Pistia stratiotes*

**DOI:** 10.1371/journal.pone.0124304

**Published:** 2015-04-17

**Authors:** Xiong Li, Yanli Zhou, Yunqiang Yang, Shihai Yang, Xudong Sun, Yongping Yang

**Affiliations:** 1 Key Laboratory for Plant Diversity and Biogeography of East Asia, Kunming Institute of Botany, Chinese Academy of Sciences, Kunming 650201, China; 2 China Germplasm Bank of Wild Species, Kunming Institute of Botany, Chinese Academy of Sciences, Kunming 650204, China; 3 University of Chinese Academy of Sciences, Beijing 100049, China; 4 Institute of Tibetan Plateau Research, Chinese Academy of Sciences, Beijing 100101, China; CSIR-National Botanical Research Institute, INDIA

## Abstract

Cadmium (Cd) pollution is an environmental problem worldwide. Phytoremediation is a convenient method of removing Cd from both soil and water, but its efficiency is still low, especially in aquatic environments. Scientists have been trying to improve the ability of plants to absorb and accumulate Cd based on interactions between plants and Cd, especially the mechanism by which plants resist Cd. *Eichhornia crassipes* and *Pistia stratiotes* are aquatic plants commonly used in the phytoremediation of heavy metals. In the present study, we conducted physiological and biochemical analyses to compare the resistance of these two species to Cd stress at 100 mg/L. *E*. *crassipes* showed stronger resistance and was therefore used for subsequent comparative proteomics to explore the potential mechanism of *E*. *crassipes* tolerance to Cd stress at the protein level. The expression patterns of proteins in different functional categories revealed that the physiological activities and metabolic processes of *E*. *crassipes* were affected by exposure to Cd stress. However, when some proteins related to these processes were negatively inhibited, some analogous proteins were induced to compensate for the corresponding functions. As a result, *E*. *crassipes* could maintain more stable physiological parameters than *P*. *stratiotes*. Many stress-resistance substances and proteins, such as proline and heat shock proteins (HSPs) and post translational modifications, were found to be involved in the protection and repair of functional proteins. In addition, antioxidant enzymes played important roles in ROS detoxification. These findings will facilitate further understanding of the potential mechanism of plant response to Cd stress at the protein level.

## Introduction

Cadmium (Cd), which is one of the most common heavy metal pollutants [1], is easily absorbed by plants and enriched in other organisms through the food chain [2,3]. Cd causes diverse biotoxic effects and diseases [2,4] that can threaten the growth, development and survival of an organism. Cd pollution has become an environmental problem worldwide because of its unseen, long-term and irreversible characteristics [5]. Thus, various methods and techniques have been developed to remove Cd and other heavy metals from the environment [6]. Among them, phytoremediation is a simple, economic and clean method that has attracted a great deal of attention [6,7].

Exposure to Cd pollution influences the development of plants in various ways. Cd can damage the cell structure and division [8], inhibit photosynthesis and transpiration [9,10], and induce oxidative stress [11,12]. However, plants can resist Cd stress through a variety of approaches. For example, they can control the absorption and use of Cd [13]. Additionally, many plants can detoxicate Cd by forming compounds with the help of phytochelatins (PCs) [14–16]. Plants can also coordinate synthesis and consumption of PCs and other thiols to form a metabolic equilibrium to accumulate Cd and combat its toxicity [17]. PCs are also considered to be important to the maintenance of glutathione and other antioxidant systems needed for plant survival [18]. Another strategy employed by plants to reduce Cd concentrations in the cytoplasm is compartmentalization [19,20], in which Cd is sequestered in vacuoles. In addition, plants can also improve antioxidant systems to respond to increased oxidative stress caused by Cd stress [21,22]. Based on these detoxification functions, some plants become hyperaccumulator species and are applied to remove Cd [23]. However, hyperaccumulator plants account for only a small part of the plant kingdom, and most plants cannot well resist Cd toxicity because of their inherent genetic basis. Nevertheless, genetic modification has been demonstrated to improve the resistance of plants to Cd [20], which may reduce Cd toxicity to plants and improve their efficiency for phytoremediation of Cd contamination. Proteomics, transcriptomics and metabolomics are able to provide accurate information regarding the molecular mechanisms of interactions between organisms and the environment [24–26]. Over the last decade, proteomic techniques have been applied to explore proteome changes induced by Cd stress in many model plants and hyperaccumulators [27–29]. Several types of functional proteins, including those involved in photosynthesis [27,30–33], energy and carbohydrate metabolism [27,29–32,34,35], transcription and translation [32,33,35], oxidation and reduction [27,29,30,33], and stress-response proteins [29,34,35], have shown common changes in most studied plants. In addition, some special proteomics have been used to investigate the targeted processes or proteins. For example, Schneider *et al*. [36] applied a quantitative proteomics approach to evaluate the contribution of vacuolar transporters to Cd detoxification in barley and identified several important transporters that might be potential candidates for further investigation. Alvarez *et al*. [37] implemented two quantitative proteomics approaches, fluorescence two-dimensional difference gel electrophoresis and multiplexed isobaria tagging technology, to demonstrate the involvement of many enzymes that played essential roles in the Cd hyperaccumation and tolerance of *Brassica juncea*. These studies helped us to better understand how plants resisted Cd or other heavy metals.

However, although hyperaccumulator plants have occasionally been discovered and the molecular mechanism of plant resistance to Cd stress has been gradually revealed, most studies conducted to date have focused on terrestrial plants [23,28], while there have been few relevant investigations of aquatic plants [23,28]; therefore, the resources used for phytoremediation of Cd-polluted water are still largely limited. It is well known that Cd can easily spread in aquatic environments as the water flows, which will lead to difficulties in management and remediation of Cd pollution in water. Thus, it is essential to identify many more aquatic Cd hyperaccumulators and improve the removal ability of Cd in common aquatic species. *Eichhornia crassipes* and *Pistia stratiotes* are two common submersed plants that have been widely applied to the remediation of sewage to reduce eutrophication and heavy metals pollution [38,39]. These two plants are known to have the attributes of rapid growth, strong resistance to pollution and being convenient for salvage [40]. Previous studies have shown that these species underwent differential accumulation effects under varying Cd concentrations [39]. However, the difference in tolerance to Cd stress between *E*. *crassipes* and *P*. *stratiotes* and the physiological and molecular mechanism through which it occurs are still unknown. In addition, previous studies were usually performed using relatively low Cd concentrations [28], while ignoring the short-term effects of high Cd concentrations on plants. Therefore, in the present study, we measured physiological and biochemical reactions to compare the resistance of *E*. *crassipes* and *P*. *stratiotes* to Cd stress at 100 mg/L. Because *E*. *crassipes* showed stronger resistance, we conducted comparative proteomics to explore the potential mechanism of *E*. *crassipes* tolerance to Cd stress. The results of this study will enhance our understanding of interactions between aquatic plants and Cd, which will improve Cd phytoremediation.

## Results

### Changes in morphology

**Fig 1:**
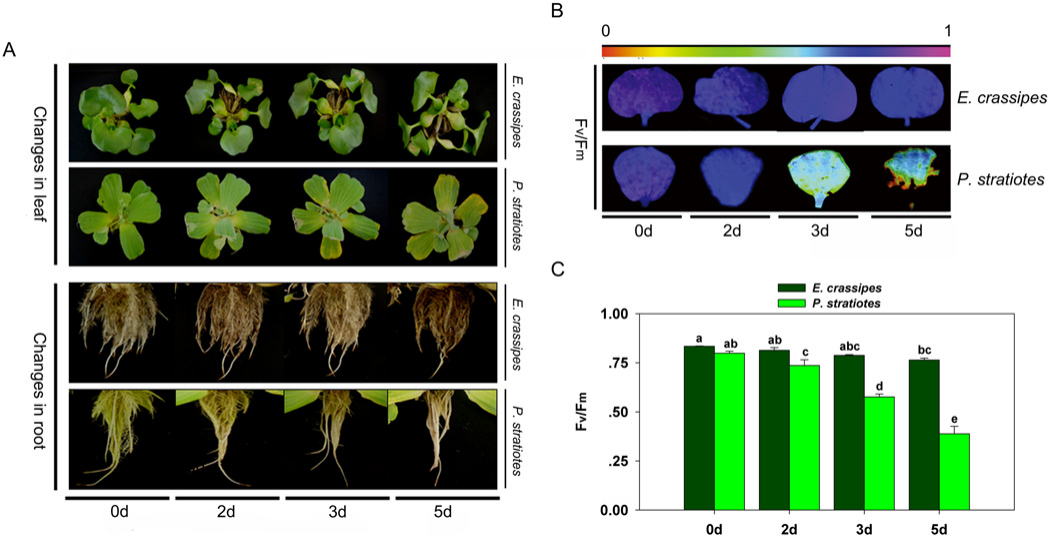
Changes in morphology and chlorophyll fluorescence of *E*. *crassipes* and *P*. *stratiotes* exposed to 100 mg/L Cd for different times. A: Changes in leaf and root morphology. B: Fv/Fm images. The pseudocolor code depicted at the top of the image ranges from 0 (red) to 1 (purple). C: Average Fv/Fm values. Data are the means ± SE. Different letters following mean values indicate significant differences (Tukey’s test, *P*<0.05).

We first observed the morphological change to see the different resistance to high-concentration Cd between *E*. *crassipes* and *P*. *stratiotes*. Under Cd stress, the leaves of *E*. *crassipes* began to droop, but showed no discoloration or withering with increased treatment time (Fig 1A). However, the leaves of *P*. *stratiotes* turned yellow and withered from the leaf edges as the treatment time increased, and they began to fall off after 5 d of treatment (Fig 1A). Greater differences were observed in the roots relative to the leaves. Specifically, the roots of *E*. *crassipes* showed no obvious changes in response to Cd exposure until 5 d of treatment (Fig 1A), at which point the lateral roots began to fall. However, the roots of *P*. *stratiotes* started falling after Cd exposure for 2 d (Fig 1A), and they became rotten after 5 d of treatment (Fig 1A).

### Changes in photosynthesis characteristics

**Fig 2:**
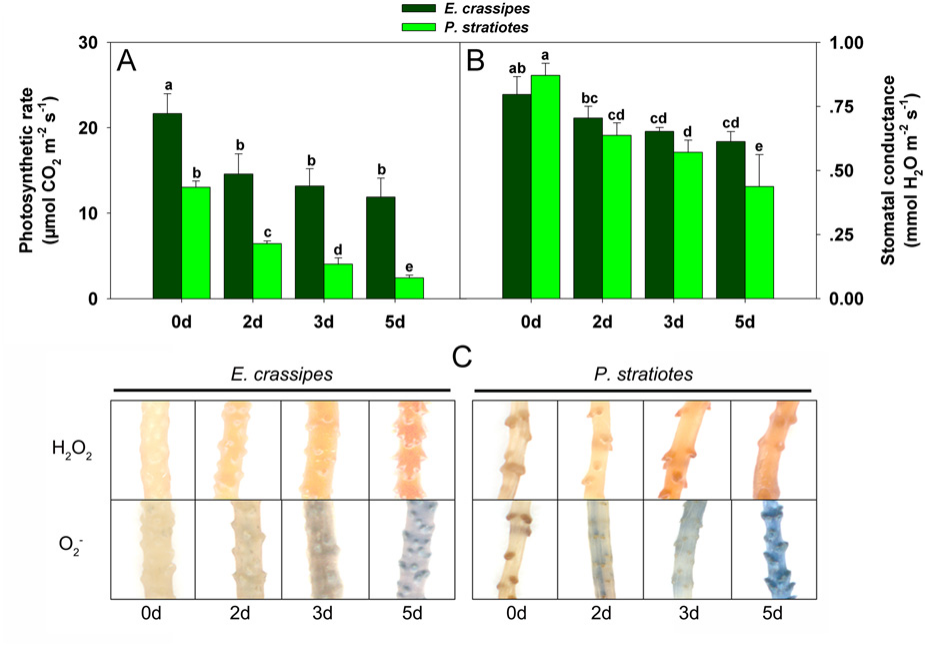
Changes in photosynthetic characteristics and reactive oxygen species (ROS) of *E*. *crassipes* and *P*. *stratiotes* exposed to 100 mg/L Cd for different times. A: Photosynthetic rate change. B: Stomatal conductance change. C: *In situ* detection of H_2_O_2_ and O_2_^-^. Data are presented as mean ± standard error. Different letters following mean values indicate significant differences (Tukey’s test, *P*<0.05).

To investigate the differential response to Cd stress between *E*. *crassipes* and *P*. *stratiotes* from the physiological level, we measured the maximum quantum yield (ratio of variable to maximum fluorescence; F_v_/F_m_) of photosystem II (PS II). In the present study, F_v_/F_m_ decreased in response to Cd treatment in both species (Fig 1B and 1C), but there were significant differences in the changes between species. Specifically, the F_v_/F_m_ of *E*. *crassipes* decreased by 2.5%, 5.7%, and 8.4% after 2, 3, and 5 d of treatment, respectively, whereas that of *P*. *stratiotes* decreased by 8.5%, 27.9%, and 51.4% relative to the corresponding controls (Fig 1C). Similarly, photosynthesis showed different reductions between species. Specifically, the photosynthetic rate and stomatal conductance were reduced in response to Cd stress in *E*. *crassipes* (Fig 2A and 2B), but no differences were observed from 2 to 5 d of treatment (Fig 2A and 2B). However, both the photosynthetic rate and stomatal conductance decreased sharply in *P*. *stratiotes* following Cd exposure, with significant differences being observed at different time points (Fig 2A and 2B).

### Reactive oxygen species (ROS), malondialdehyde (MDA) and proline accumulation

**Fig 3:**
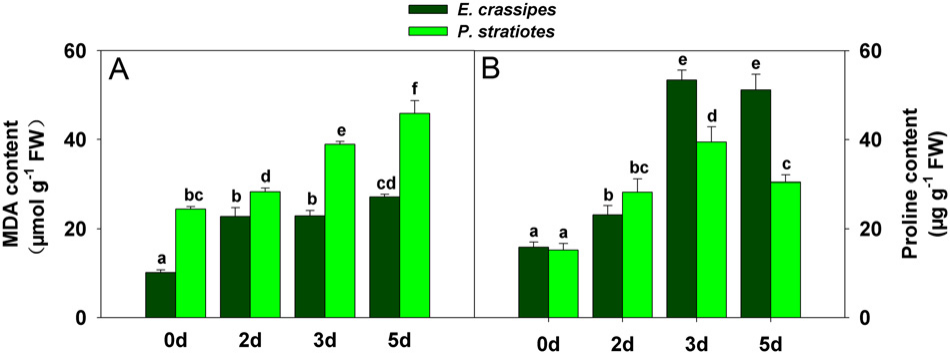
Changes in malondialdehyde (MDA) and proline content in *E*. *crassipes* and *P*. *stratiotes* exposed to 100 mg/L Cd for different times. A: MDA content change. B: Proline content change. Data are presented as mean ± standard error. Different letters following mean values indicate significant differences (Tukey’s test, *P*<0.05).

To understand the change of ROS metabolism in plants under Cd stress, we detected the accumulation of ROS (H_2_O_2_ and O_2_^-^) and MDA. H_2_O_2_ and O_2_^-^ both increased gradually with increasing treatment time in *E*. *crassipes* and *P*. *stratiotes* (Fig 2C). *P*. *stratiotes* obviously produced more H_2_O_2_ and O_2_^-^ after the same treatment time when compared with *E*. *crassipes* (Fig 2C). Similarly, the level of MDA increased gradually in both *E*. *crassipes* and *P*. *stratiotes* with increasing Cd exposure duration, but with different accumulation levels between species (Fig 3A). The MDA content in *E*. *crassipes* increased rapidly at first, then continued to increase slightly, whereas it increased rapidly throughout the experimental period in *P*. *stratiotes* (Fig 3A).

The proline content was measured to explore the potential role of proline in response to Cd stress. In our study, the proline content differed between *E*. *crassipes* and *P*. *stratiotes* (Fig 3B). Although the initial proline content in *E*. *crassipes* was less than in *P*. *stratiotes*, it continued to increase with exposure time (Fig 3B). Conversely, the proline content in *P*. *stratiotes* increased from 2 to 3 d of treatment, but decreased at 5 d (Fig 3B).

### Dynamics of antioxidant enzyme activities

**Fig 4:**
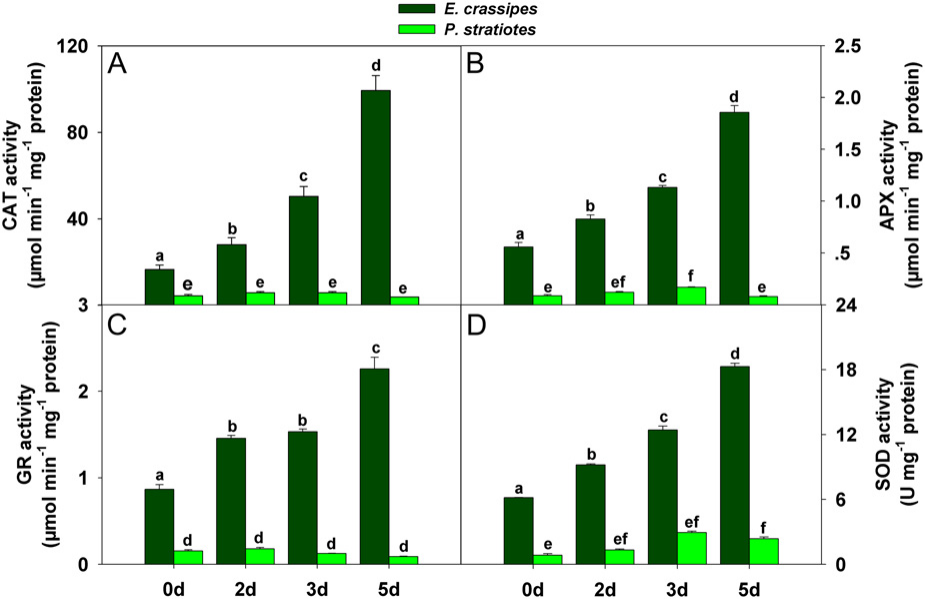
Changes in antioxidant enzyme activity in *E*. *crassipes* and *P*. *stratiotes* exposed to 100 mg/L Cd for different times. A: CAT activity. B: APX activity. C: GR activity. D: SOD activity. Data are presented as mean ± standard error. Different letters following mean values indicate significant differences (Tukey’s test, *P*<0.05).

To investigate the role of antioxidant system in regulating ROS accumulation, we measured the activities of four common antioxidant enzymes. The activities of catalase (CAT; EC 1.11.1.6), ascorbate peroxidase (APX; EC 1.11.1.11), glutathione reductase (GR; EC 1.8.1.7), and superoxide dismutase (SOD; EC 1.15.1.1) in *E*. *crassipes* were consistently much higher than in *P*. *stratiotes* (Fig 4), and they all increased significantly with increasing treatment time in *E*. *crassipes* (Fig 4). However, antioxidant enzyme activities first increased, then decreased in *P*. *stratiotes* (Fig 4). The maximum CAT, APX, and SOD activities were observed at 3 d, after which they began to decrease (Fig 4A, 4B and 4D). The highest GR activity was observed following exposure to Cd stress for 2 d (Fig 4C).

### Dynamic change in expression of differential proteins

**Fig 5:**
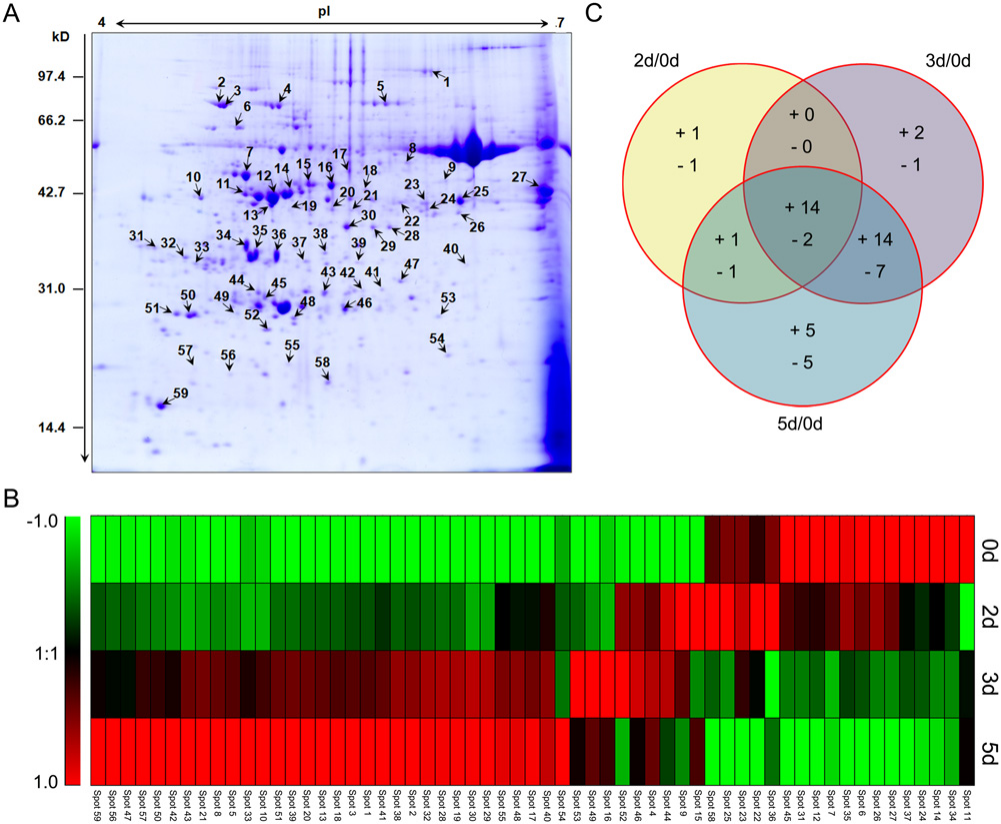
Comparative proteomics analyses of four *E*. *crassipes* samples treated with 100 mg/L Cd for different times. A: Representative 2-DE (the control sample of the first set of gel images) showing spot numbers of identified proteins. B: Hierarchical clustering of the identified protein expression profiles of different samples. Different colors correspond to the protein log-transformed fold-change ratios depicted in the bar on the left of the figure. C: Venn diagram analysis of differentially expressed proteins of each treated sample compared with the control sample (0d). “+” and “-” represent up-regulated and down-regulated proteins, respectively.

To further explore the underlying mechanism of *E*. *crassipes* tolerance toward Cd stress, the leaf proteomes of *E*. *crassipes* samples were evaluated by two-dimensional electrophoresis (2-DE). Each sample was replicated three times (S1, S2 and S3 Figs), and more than 500 protein spots were detected within each sample after staining. Of these, 87 showed increased expression (>1.50) or decreased expression (<0.67) in treated samples (2–5d) relative to the control (0d). Ultimately, 59 differentially expressed proteins (Fig 5A) were successfully identified by MALDI-TOF-MS/MS analysis and the National Center for Biotechnology Information (NCBI) nonredundant protein database (Table 1). Hierarchical cluster analysis was conducted to categorize the identified proteins that showed differential expression profiles in response to Cd stress (Fig 5B). Venn diagram analysis was used to reflect change patterns in proteins from treated samples (2–5d) relative to the control (0d) (Fig 5C). The results showed that up-regulation was much greater than down-regulation (Fig 5C), and that proteins were mainly affected during the later stage (3 or 5d) of Cd stress (Fig 5C).

**Table 1:** Identification and analysis of differentially expressed proteins in leaves of *Eichhornia crassipes* treated by Cd stress for different times.

Spot	Protein name	Acc. No.^a^	Theo. Mw/pI^b^	Exp. Mw/pI^c^	SC^d^	Score	Organism	Ratio^e^		
								2d/0d	3d/0d	5d/0d
**Photosynthesis**										
6	putative rubisco subunit binding-protein alpha subunit precursor (60 kDa chaperonin alpha subunit)	gi|31193919	61.36/5.36	68.71/5.05	29.11	118	*Oryza sativa Japonica Group*	0.87	0.72	0.54
7	predicted ribulose bisphosphate carboxylase/oxygenase activase 1, chloroplastic-like	gi|359481752	52.19/5.69	57.14/5.11	14.77	394	*Vitis vinifera*	0.75	0.48	0.45
12	ribulose-1,5-bisphosphate carboxylase/oxygenase activase 1	gi|12620881	48.19/5.54	38.71/5.30	19.63	334	*Gossypium hirsutum*	0.75	0.64	0.49
14	RuBisCO activase	gi|445628	42.95/5.50	40.17/5.43	12.79	115	*Nicotiana tabacum*	0.70	0.59	0.52
24	fructose-bisphosphate aldolase, chloroplast precursor, putative, expressed	gi|108864048	41.81/6.07	39.74/6.34	24.22	370	*Oryza sativa Japonica Group*	0.60	0.56	0.42
26	chloroplast stem-loop binding protein-41	gi|15229384	44.07/8.54	38.46/6.54	14.25	141	*Arabidopsis thaliana*	0.90	0.66	0.52
34	chloroplast photosynthetic water oxidation complex 33kDa subunit precursor	gi|152143640	28.48/5.48	41.99/5.12	22.64	159	*Morus nigra*	0.44	0.34	0.28
35	OEE1	gi|302595735	34.49/5.40	39.98/5.19	30.86	435	*Helianthus annuus*	0.92	0.72	0.48
45	predicted carbonic anhydrase, chloroplastic-like	gi|357130587	51.29/8.90	34.35/5.67	21.57	57	*Brachypodium distachyon*	0.82	0.66	0.55
52	thylakoid luminal 19 kDa protein	gi|357457687	26.38/5.82	27.09/5.27	16.94	96	*Medicago truncatula*	1.17	1.55	1.03
59	ribulose bisphosphate carboxylase	gi|119720808	18.71/8.23	17.89/6.84	37.65	99	*Brassica rapa*	1.47	1.72	2.56
**Growth and development**										
5	maturase K	gi|197257987	57.74/9.86	78.01/6.02	17.55	40	*Siphocodon spartioides*	1.12	1.39	1.66
15	actin	gi|218533930	41.88/5.31	42.44/5.55	55.97	801	*Caragana korshinskii*	2.22	1.25	1.69
18	old-yellow-enzyme homolog	gi|2232254	42.13/5.90	41.23/5.94	11.08	132	*Catharanthus roseus*	1.70	2.30	3.11
**Metabolism processes**										
**Biosynthesis and degradation**										
16	glutamine synthetase	gi|15238559	47.78/6.43	41.86/5.72	15.35	191	*Arabidopsis thaliana*	1.10	1.53	2.04
22	beta-cyanoalanine synthase	gi|30840956	38.26/6.38	40.62/6.13	6.53	75	*Betula pendula*	1.14	0.88	0.66
23	malate dehydrogenase	gi|320449084	35.79/5.76	39.95/6.30	25.9	176	*Zea mays*	1.06	0.95	0.64
27	predicted aminomethyl transferase, mitochondrial-like	gi|356555678	44.72/8.77	43.51/6.97	28.01	85	*Glycine max*	0.97	0.76	0.53
37	xyloglucantransglusylase/hydrolase 1	gi|304273280	32.13/6.06	39.46/5.53	3.93	69	*Gladiolus grandiflorus*	0.88	0.77	0.62
44	cytosolic triosephosphate isomerase	gi|310768740	27.26/5.04	34.51/5.21	13.04	67	*Pteris vittata*	1.31	1.82	2.32
47	triosephosphate isomerase, cytosolic	gi|226495391	27.24/5.52	35.11/6.24	29.25	87	*Zea mays*	1.17	1.27	1.70
58	granule-bound starch synthase precursor	gi|4588607	63.39/7.86	15.78/5.80	51.68	772	*Triticum aestivum*	1.28	0.77	0.52
**Energy related**										
11	AMP deaminase family protein	gi|566209963	90.88/6.30	40.03/5.11	10.36	51	*Populus trichocarpa*	0.61	0.79	0.81
40	ribulose-1,5-bisphosphate carboxylase/oxygenase large subunit	gi|148787961	48.36/6.34	40.02/6.62	45.6	685	*Eichhornia crassipes*	2.33	2.70	2.88
41	predicted probable ATP synthase 24 kDa subunit, mitochondrial	gi|225438529	27.67/9.26	36.26/6.05	17.01	90	*Vitis vinifera*	2.05	2.60	3.27
56	ATP synthase CF1 epsilon subunit (chloroplast)	gi|374257035	14.69/4.95	20.26/5.03	53.73	466	*Japonolirion osense*	1.17	1.28	1.71
**Oxidation-reduction process**										
1	glycine dehydrogenase, putative	gi|255550796	115.78/6.57	108.69/6.37	8.8	126	*Ricinus communis*	1.74	2.29	3.04
8	glycine dehydrogenase, putative	gi|255580957	42.88/6.05	45.19/6.23	36.97	611	*Ricinus communis*	1.28	1.77	2.19
19	plastidic aldolase	gi|164470331	43.16/6.38	38.24/5.43	35.52	229	*Solanum tuberosum*	2.16	3.80	4.30
20	plastidic aldolase family protein	gi|224094919	42.72/6.85	38.56/5.72	37.37	67	*Populus trichocarpa*	1.50	1.99	2.44
25	predicted glyceraldehyde-3-phosphate dehydrogenase A, chloroplastic-like	gi|357163943	43.11/7.01	41.09/6.55	8.44	100	*Brachypodium distachyon*	1.25	0.61	0.43
28	isoflavone reductase-like protein	gi|373939378	33.29/5.74	35.81/6.10	4.25	88	*Daucus carota*	1.73	2.78	3.06
29	phenylcoumaran benzylic ether reductase	gi|3114899	33.99/5.66	35.62/5.99	14.94	86	*Populus trichocarpa*	2.77	3.43	3.91
30	pterocarpan reductase	gi|116077986	33.97/5.94	45.60/5.82	6.13	89	*Lotus japonicus*	1.36	2.93	3.20
36	oxidoreductase, aldo/keto reductase family protein, expressed isoform 1	gi|590718087	39.48/7.56	39.74/5.33	27.24	52	*Theobroma cacao*	1.16	0.57	0.76
46	peptide methionine sulfoxide reductase	gi|357494493	22.92/5.84	32.97/5.82	17.82	95	*Medicago truncatula*	1.77	1.88	1.57
**Defense response**										
2	chloroplast heat shock protein 70–1	gi|15233779	76.58/5.07	76.57/4.93	14.35	281	*Arabidopsis thaliana*	1.55	2.05	2.34
3	heat shock protein, putative	gi|255570990	75.43/5.35	76.45/4.97	15.79	612	*Ricinus communis*	1.64	2.10	2.67
4	70 kDa heat shock cognate protein 2	gi|45331283	71.58/5.14	76.65/5.34	34.41	622	*Vigna radiata*	2.03	2.20	2.04
33	14-3-3 family protein	gi|55375985	29.79/4.75	40.02/4.75	29.77	167	*Malus x domestica*	1.02	1.61	2.48
42	2-oxoglutarate-iron(II) dependent oxygenase	gi|302815609	36.85/5.77	35.96/5.93	17.43	39	*Selaginella moellendorffii*	1.17	1.52	2.19
**Antioxidant enzymes**										
43	cytosolic ascorbate peroxidase	gi|153799884	27.95/5.16	35.19/5.67	22.71	189	*Dimocarpus longan*	1.13	1.86	2.37
51	2-cys-peroxiredoxin	gi|327422155	22.21/4.92	30.89/4.59	18.59	303	*Vigna unguiculata*	1.23	1.51	1.75
55	chloroplast copper/zinc superoxide dismutase	gi|304651504	20.38/5.31	20.15/5.38	13.93	191	*Hordeum vulgare*	2.07	2.44	2.76
**Ion transport and regulation**										
21	cation efflux family protein isoform 2	gi|590613599	45.81/5.76	39.07/5.88	33.25	41	*Theobroma cacao*	1.07	1.28	1.42
48	calcineurin B-like protein	gi|357437489	28.33/4.68	31.18/5.46	6.25	46	*Medicago truncatula*	1.46	1.68	1.81
**Transcription and translation**										
9	elongation factor tu, putative	gi|255567660	49.29/6.62	43.25/6.46	23.83	120	*Ricinus communis*	1.69	1.42	1.15
10	peptidyl-prolyl cis-trans isomerase, putative	gi|255552604	51.55/4.97	39.73/4.78	17.63	71	*Ricinus communis*	1.12	1.91	2.79
17	chloroplast translational elongation factor Tu	gi|6525065	50.55/6.05	42.76/5.84	27.62	370	*Oryza sativa Japonica Group*	1.47	1.66	1.85
31	nucleic acid binding protein1	gi|162463757	33.15/4.60	40.55/4.33	21.77	170	*Zea mays*	0.84	0.74	0.66
32	putative elongation factor	gi|90704791	24.69/4.56	41.07/4.65	10.62	112	*Cryptomeria japonica*	1.34	1.76	1.89
39	DNA-binding storekeeper protein-related transcriptional regulator	gi|18411272	34.04/5.84	36.23/5.85	18.45	42	*Arabidopsis thaliana*	1.21	1.45	1.66
54	small ribosomal protein subunit 4	gi|67035885	21.91/9.91	23.29/6.21	21.52	61	*Pterogonidium pulchellum*	1.04	1.02	1.51
**Protein post-translational modification**										
38	predicted phosphoglycolate phosphatase-like	gi|357164381	39.01/5.76	40.94/5.66	14.21	107	*Brachypodium distachyon*	1.47	1.99	2.23
49	predicted methyltransferase-like protein 23-like isoform X1	gi|568825272	27.17/5.06	31.59/5.02	6.87	41	*Citrus sinensis*	1.10	1.50	1.31
57	ubiquitin-like superfamily protein	gi|145360542	27.71/6.33	22.99/4.74	23.67	46	*Arabidopsis thaliana*	1.14	1.30	1.56
**Others**										
13	ALA-interacting subunit 5	gi|42572169	32.05/9.28	35.54/5.32	22.26	49	*Arabidopsis thaliana*	1.34	1.65	1.98
50	zinc knuckle family protein	gi|357498441	41.47/8.56	30.54/4.68	48.5	43	*Medicago truncatula*	1.12	1.29	1.57
53	AP3-2 type 1	gi|27990434	22.45/8.98	26.09/6.48	27.98	58	*Berberis gilgiana*	1.22	1.83	1.38

^a^Acc. No., database accession numbers according to NCBInr;

^b^Theo. Mw/pI, theoretical Mw/pI;

^c^Exp. Mw/pI, experimental Mw/pI;

^d^SC, sequence coverage;

^e^Ratio, different protein spot intensity ratios of samples after 2 d, 3 d and 5 d exposure relative to the control (0d).

### Functional classification of identified proteins

**Fig 6:**
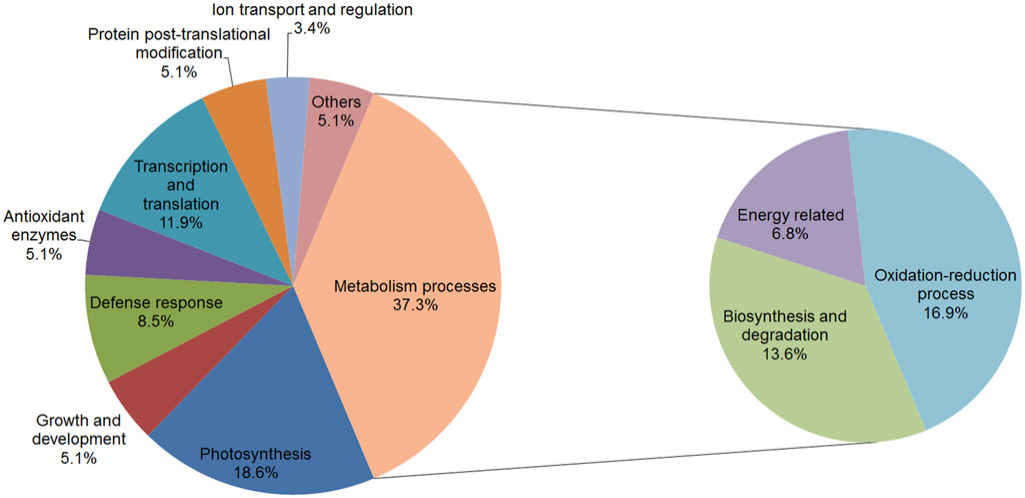
Functional classification of the identified proteins.

The identified proteins could be classified into nine functional groups: photosynthesis [putative rubisco subunit binding-protein alpha subunit precursor (spot 6), predicted ribulose bisphosphate carboxylase/oxygenase activase 1, chloroplastic-like (spot 7), ribulose-1,5-bisphosphate carboxylase/oxygenase activase 1 (spot 12), RuBisCO activase (spot 14), fructose-bisphosphate aldolase, chloroplast precursor, putative, expressed (spot 24), chloroplast stem-loop binding protein-41 (spot 26), chloroplast photosynthetic water oxidation complex 33kDa subunit precursor (spot 34), OEE1 (spot 35), predicted carbonic anhydrase, chloroplastic-like (spot 45), ribulose bisphosphate carboxylase (spot 59), and thylakoid luminal 19 kDa protein (spot 52)], growth and development [maturase K (spot 5), actin (spot 15), and old-yellow-enzyme homolog (spot 18)], metabolism processes, defense response [chloroplast heat shock protein 70–1 (spot 2), putative heat shock protein (spot 3), and 70 kDa heat shock cognate protein 2 (spot 4), 14-3-3 family protein (spot 33), and 2-oxoglutarate-iron(II)-dependent oxygenase (spot 42)], antioxidant enzymes [cytosolic ascorbate peroxidase (spot 43), 2-cys-peroxiredoxin (spot 51), and chloroplast copper/zinc superoxide dismutase (spot 55)], ion transport and regulation [cation efflux family protein isoform 2 (spot 21) and calcineurin B-like protein (spot 48)], transcription and translation [elongation factor tu, putative (spot 9), putative peptidyl-prolyl cis-trans isomerase (spot 10), chloroplast translational elongation factor Tu (spot 17), nucleic acid binding protein1 (spot 31), putative elongation factor (spot 32), DNA-binding storekeeper protein-related transcriptional regulator (spot 39), and small ribosomal protein subunit 4 (spot 54)], protein post-translational modification [predicted phosphoglycolate phosphatase-like (spot 38), predicted methyltransferase-like protein 23-like isoform X1 (spot 49), and ubiquitin-like superfamily protein (spot 57)] and proteins with other functions [ALA-interacting subunit 5 (spot 13), zinc knuckle family protein (spot 50), and AP3-2 type 1 (spot 53)] (Table 1). In particular, the proteins involved in metabolism processes could be further divided into three categories: biosynthesis and degradation [Glutamine synthetase (spot 16), beta-cyanoalanine synthase (spot 22), malate dehydrogenase (spot 23), predicted aminomethyl transferase, mitochondrial-like (spot 27), xyloglucantransglusylase/hydrolase 1 (spot 37), cytosolic triosephosphate isomerase (spot 44), triosephosphate isomerase, cytosolic (spot 47), and granule-bound starch synthase precursor (spot 58)], energy related [AMP deaminase family protein (spot 11), ribulose-1,5-bisphosphate carboxylase/oxygenase large subunit (spot 40), predicted probable ATP synthase 24 kDa subunit, mitochondrial (spot 41), and ATP synthase CF1 epsilon subunit (chloroplast) (spot 56)] and oxidation-reduction process [glycine dehydrogenase, putative (spot 1), glycine dehydrogenase, putative (spot 8), plastidic aldolase (spot 19), plastidic aldolase family protein (spot 20), predicted glyceraldehyde-3-phosphate dehydrogenase A, chloroplastic-like (spot 25), isoflavone reductase-like protein (spot 28), phenylcoumaran benzylic ether reductase (spot 29), pterocarpan reductase (spot 30), oxidoreductase, aldo/keto reductase family protein, expressed isoform 1 (spot 36), and peptide methionine sulfoxide reductase (spot 46)] (Table 1). Among all the identified proteins, the functional group of metabolism processes (37.3%) accounted for the largest number of differentially expressed proteins (Fig 6). In addition, proteins related to photosynthesis (18.6%), transcription and translation (11.9%), and defense response (8.5%) also constituted larger proportions of the differential proteins (Fig 6).

## Discussion

### Differences in tolerance of Cd between *E*. *crassipes* and *P*. *stratiotes*

*E*. *crassipes* and *P*. *stratiotes* are often used together in studies of heavy metals pollution [39,41], and these organisms commonly exhibit different accumulation effects when exposed to Cd^2+^, Zn^2+^, and Cu^2+^ [39]. However, less work has focused on the toxicity of Cd toward these plants, or on their Cd resistance. In this study, we compared the tolerance of Cd stress between *E*. *crassipes* and *P*. *stratiotes* based on morphological and physiological aspects. When exposed to Cd for the same time period, the leaves and roots of *P*. *stratiotes* were more seriously damaged than those of *E*. *crassipes*, with significant yellowing of the leaves and shedding of roots occurring (Fig 1A). Physiological detection showed that photosynthesis was greatly inhibited in *P*. *stratiotes* relative to *E*. *crassipes* (Fig 2A). *P*. *stratiotes* also suffered more severe oxidative stress or damage owing to a higher generation of ROS (Fig 2C) and their oxidation product, MDA (Fig 3A). These results demonstrated that *E*. *crassipes* was much more tolerant to Cd than *P*. *stratiotes*.

*E*. *crassipes* has long been regarded as one of the best plants for sewage purification and is widely applied in ecological restoration engineering [42] based on its strong tolerance of sewage and excellent growth characteristics [40]. In this study, *E*. *crassipes* appeared to be able to normalize its physiological functions, even after being subjected to 100 mg/L Cd, indicating its strong resistance to contamination and potential for application in removal of Cd from water. To further understand the underlying mechanisms of Cd tolerance, proteomics analysis of *E*. *crassipes* was conducted in conjunction with biochemical analyses during exposure to Cd.

### Proteins involved in photosynthesis

As shown in Figs 1B, 1C and 2A, inhibited photosynthesis is one of the most obvious phenomena in plants stressed by Cd [10,43]. It is generally believed that this occurs in response to blockage of photosynthetic pigment synthesis [43] and destruction of chloroplasts [8]. These effects could result in leaf chlorosis and wither, which were shown in our study (Fig 1A). Many studies have shown that proteins related to photosynthesis were commonly influenced by Cd stress [27,30–33]. In the present study, the photosystem efficiency of both *E*. *crassipes* and *P*. *stratiotes* decreased (Figs 1C, 2A and 2B) as observed in other plants exposed to Cd [30,31]. Accordingly, most proteins involved in photosynthesis (spots 6, 7, 12, 14, 24, 26, 34, 35, and 45) were down-regulated in *E*. *crassipes* with increasing treatment time (Fig 5B and Table 1). The down-regulation of these proteins has often been observed in other studies of the effects of Cd stress on plants [33]. For example, Rubiso, a key enzyme involved in CO_2_ assimilation during the Calvin—Benson cycle, has been reported to be compromised by Cd in both non-hyperaccumulator [31,44] and hyperaccumulator plants [33,45]. The results of the present study indicated that the efficiency of CO_2_-fixation decreased under Cd stress, which confirmed the reduction in photosynthesis. However, several proteins were induced by Cd exposure. For example, a photosynthetic enzymes, ribulose bisphosphate carboxylase (spot 59), showed continuously increasing expression, while thylakoid luminal 19 kDa protein (spot 52) was up-regulated during the early stage (3 d) and then decreased to the control level (5 d) (Fig 5B and Table 1). These results suggested that the stimulation of some proteins played essential roles in maintenance of the photosynthesis when other proteins were inhibited.

### Proteins involved in metabolism processes

Relevant proteomics results have indicated that exposure to Cd resulted in the alteration of proteins related to biosynthesis and degradation [27,31,34,35]. The proteins involved in different metabolic pathways usually exhibit diverse expression patterns during Cd treatment [27,31,34,35]. In the present study, two proteins related to biosynthesis (spots 27 and 37) were continuously down-regulated as the treatment time increased (Fig 5B and Table 1). Additionally, three related proteins (spots 22, 23, and 58) were not down-regulated until exposure to Cd for 3 d (Fig 5B and Table 1). However, some enzymes in metabolic pathways often showed up-regulation under Cd stress. Glutamine synthetase (GS) is involved in the synthesis of glutathione (GSH) via the glutamate biosynthesis pathway [32], and GSH can synthesize PCs via PC synthase, which can form complexes with Cd in cytosol and then be transported into vacuoles [46]. Triosephosphate isomerase, a key enzyme in glycolysis, plays an important role in efficient energy production [27]. These enzymes showed increased abundance in studies of exposure to Cd in soybean [27] and *B*. *juncea* [30], which was consistent with the results of the present study. Glutamine synthetase (spot 16), cytosolic triosephosphate isomerase (spot 44) and triosephosphate isomerase, cytosolic (spot 47) showed increasing expression during exposure to Cd stress in our study (Fig 5B and Table 1). Overall, the results indicated that *E*. *crassipes* had an active response to Cd stress, even if some biosynthesis pathways were restrained.

Proteins involved in energy metabolism have been confirmed to play important roles in plant response to abiotic stresses [47–49]. When exposed to Cd stress, plants were shown to increase energy demand, so proteins related to energy production, such as ATP synthetase, usually showed enhanced abundance [27,31,33]. In the present study, we identified four proteins related to energy metabolism (Table 1). Three of these proteins (spots 40, 41, and 56) exhibited increasing expression, while the expression of only one protein (spot 11) decreased as the treatment time increased (Fig 5B and Table 1). These results demonstrate the important role of energy in plant response to stress.

In addition, ten proteins related to the oxidation-reduction process (spots 1, 8, 19, 20, 25, 28, 29, 30, 36, and 46) exhibited various expression patterns among samples exposed to Cd for different lengths of time (Fig 5B and Table 1). The differential expression of some of these proteins has been reported in previous studies [28], while that of others is reported here for the first time. Their diverse expression indicated that they actively coped with or were passively affected by Cd stress.

### Protein synthesis and modification

Dramatic changes in a number of proteins associated with transcription and translation have been observed in numerous plants under Cd stress [33,35]. Different from some previous studies [33,35], most proteins involved in transcription and translation (spots 10, 17, 32, 39, and 54) were gradually up-regulated in the present study. One protein (spot 31) was down-regulated and one protein (spot 9) was up-regulated in the early stage and down-regulated in the later stage during application of Cd stress (Fig 5B and Table 1). The various expression patterns suggested that proteins related to transcription and translation played diverse roles in the treatment of Cd stress.

Protein post-translation modifications such as ubiquitination, phosphorylation, and methylation play very important roles in organisms [50]. These changes can result in the protein structure becoming more complex and the function being improved, which results in more precise adjustments and more specific effects [50]. One important physiological function of cells regulated by protein post-translation modifications is the cellular response to environmental conditions [50]. For example, protein phosphorylation is considered to be closely related to the interaction between *Kobresia pygmaea* and the environment with increasing elevation [47]. A proteomics study of the hyperaccumulator plant *Phytolacca Americana* by Zhao *et al*. [33] indicated that post-translational modifications such as phosphorylation might have occurred during Cd treatment. In the present study, we found that predicted phosphoglycolate phosphatase-like (spot 38), predicted methyltransferase-like protein 23-like isoform X1 (spot 49), and ubiquitin-like superfamily protein (spot 57), which are related to phosphorylation, methylation, and ubiquitination, respectively, showed consistent up-regulation under Cd stress (Fig 5B and Table 1). These findings indicated that post-translation modifications might participate in the regulation of *E*. *crassipes* resistance to Cd stress.

### Antioxidant enzymes and related proteins

An obvious response of plants to Cd exposure is oxidative stress caused by ROS [27,29,30,33]. Many studies have demonstrated that plant antioxidant systems can be induced to eliminate excessive ROS and prevent oxidation [21,22]. However, previous studies have also revealed that the antioxidant systems in different plant species are usually quite different [49], and high-concentration Cd stress may inhibit plant antioxidant systems [11,51]. Similar results were observed upon comparison of *E*. *crassipes* and *P*. *stratiotes* (Fig 4). The differences in activities of CAT, APX, GR and SOD between *E*. *crassipes* and *P*. *stratiotes* showed that *E*. *crassipes* had stronger antioxidant ability than *P*. *stratiotes*. Thus, higher levels of ROS (H_2_O_2_ and O_2_^-^) accumulated in *P*. *stratiotes* than *E*. *crassipes* with increasing Cd exposure time (Fig 2C), which led to the generation of high concentrations of MDA (Fig 3A). The enzymes involved in oxidative stress defenses also show dynamic expression in plants under Cd stress [28]. Proteomic-related studies revealed that the enzymes involved in peroxide detoxification [31,37,52,53] and peroxiredoxins [53–55] were usually upregulated by Cd in plants. Similarly, cytosolic ascorbate peroxidase (spot 43) and 2-cys-peroxiredoxin (spot 51) were differentially up-regulated in *E*. *crassipes* with increasing Cd exposure time (Fig 5B and Table 1). Cu/Zn SOD were downregulated in several plants under Cd stress [31,37,56], but the chloroplast copper/zinc superoxide dismutase (spot 55) in our study showed increased abundance during Cd treatment. In summary, common and unique changes in the expression of enzymes related to ROS detoxification were observed in *E*. *crassipes* when compared with other plants, indicating their important roles in protecting cell structure and function.

### Proline and proteins involved in defense response

Proline can protect plant cells against several stresses during various stages of accumulation [57], which helps plants avoid oxidative damage [58] by mediating osmotic adjustment and stabilizing macromolecules [59]. Proline also has been reported to be induced by Cd in *Silene vulgaris* [60], and the accumulation was proposed to be a consequence of metal-induced water deficit [60]. Based on the previous studies, we measured the proline content in our study. The results clearly demonstrated that proline could be induced by Cd; however, the accumulation mechanism requires further study. The different change in proline content between *E*. *crassipes* and *P*. *stratiotes* revealed differential resistance to Cd treatment by these two species.

Stress-related proteins have been shown to play an essential role in plant resistance to Cd stress [29,34,35]. HSPs are an important group of protective proteins that can protect other proteins from damage or repair damaged proteins [61]. HSPs can accumulate when plants are exposed to various stresses, including Cd treatment [31,53,54,62,63]. In the present study, three HSPs in *E*. *crassipes*, chloroplast heat shock protein 70–1 (spot 2), putative heat shock protein (spot 3), and 70 kDa heat shock cognate protein 2 (spot 4), were all obviously up-regulated as the treatment time of Cd stress increased (Fig 5B and Table 1), indicating that they played significant roles in tolerance to Cd stress. 14-3-3 proteins are known to participate in the regulation of plant development and stress responses in higher plants [64]. For example, a 14-3-3 protein in tomato modulates H^+^ efflux, basipetal auxin transport, and the PKS5-J3 pathway during root growth following alkaline stress [65]. The results of the present study showed that a 14-3-3 family protein (spot 33) was up-regulated with increased treatment time (Fig 5B and Table 1), indicating its importance to the response to Cd stress. This is first study to report induction of this protein in response to Cd stress in plants. Interestingly, we also found that a 14-3-3 protein showed induced expression in *E*. *crassipes* cultured in the eutrophic water [66]. These findings appeared to indicate the unique function of 14-3-3 proteins in response to sewage in *E*. *crassipes*. In addition, another protein (spot 42) was also up-regulated during exposure to Cd stress (Fig 5B and Table 1). Taken together, these findings suggest that the metabolites and proteins involved in resistance to stress helped *E*. *crassipes* tolerate high levels of Cd.

### Proteins involved in ion transport and regulation

The absorption, transportation, or discharge of Cd^2+^ in plants is a complex sequence of processes regulated by various transporters [67–70]. Schneider *et al*. [36] specifically employed quantitative proteomics to elucidate the contribution of vacuolar transporters in barley subjected to Cd treatment. Ultimately, they identified 56 vacuolar transporters with various expression patterns and demonstrated that some played important roles in Cd detoxification [36]. In the present study, two proteins related to ion transport (spots 21 and 48) were up-regulated in *E*. *crassipes* with increasing Cd exposure (Fig 5B and Table 1), suggesting that they played a significant role in ameliorating Cd stress.

## Conclusion

In the present study, *E*. *crassipes* exhibited stronger tolerance to high-concentration Cd stress than *P*. *stratiotes* at the morphological and physiological level; therefore, we performed comparative proteomics to explore the internal mechanism of the *E*. *crassipes* response to Cd stress. Based on the functional categories and expression patterns of 59 differential proteins, we identified a series of complex regulation processes during the response to Cd stress. While some proteins involved in life activities were negatively restrained, analogous proteins were up-regulated to compensate for the corresponding functions. Thus, *E*. *crassipes* could still maintain a higher physiological status relative to *P*. *stratiotes*. At the same time, several stress-resistance substances and proteins including proline and HSPs, as well as protein post-translational modifications, were found to be involved in the protection and renovation of functional proteins. In addition, antioxidant enzymes played important roles in the removal of excess ROS to reduce oxidative stress. These findings will lead to improved understanding of the potential mechanism of plant responses to Cd stress at the protein level.

## Materials and Methods

### Ethics statement

Plant materials used in this study were collected from Lake Dianchi (N 25°01′38″, E 102°40′21″) in Kunming, Yunnan Province, China. No specific collecting permits were required for this location. We confirm that the plants we used are neither endangered nor protected species.

### Material collection and treatment

Both *E*. *crassipes* and *P*. *stratiotes* plantlets were collected from Lake Dianchi (N 25°01′38″, E 102°40′21″) in May 2013 during the clonal reproduction period. Samples were collected from the same population so that they would have a similar genetic background. The plantlets were acclimated in a greenhouse (sunlight; 25–28°C/18–20°C, 12h-day/12h-night) for 15 d using Hoagland’s nutrient solution (HNS) [71] as the planting water. Isometric plantlets of *E*. *crassipes* and *P*. *stratiotes* were then placed into water boxes (30 cm × 20 cm × 20 cm). Each plantlet was treated in a single box with 10 L HNS water added by 100 mg/L CdCl_2_ in the greenhouse (sunlight; 25–28°C/18–20°C, 12h-day/12h-night). The plantlets were then photographed and collected for subsequent measurement and analysis at 0, 2, 3, and 5 d, respectively. There were three replicates for each time point sample.

### Chlorophyll fluorescence and photosynthetic measurement

Chlorophyll fluorescence was analyzed as previously described [49] using a pulse-amplitude modulation chlorophyll fluorometer (Heinz Walz GmbH, Effeltrich, Germany). Briefly, *E*. *crassipes* were dark-adapted for 30 min at the time of sampling to measure the maximum quantum yield (F_v_/F_m_) of photosystem II (PSII) by analyzing a whole leaf. The maximum fluorescence (F_m_) was recorded by a 0.8-s pulsed light of 4,000 μmol s^-1^ m^-2^, while the minimal fluorescence (F_o_) was recorded during the weak measuring pulses.

A portable photosynthesis system (Li-6400; Li-Cor Inc., Lincoln, NE, USA) was used to measure the net photosynthetic rate and stomatal conductance of leaves. During the measurements, the water vapor pressure deficit was set to about 1.0 kPa and the atmospheric CO_2_ concentration was 400 μmol mol^-1^. The leaf was illuminated by either a quartz halogen light source or a red light-emitting diode (Li-6400-02, Li-Cor Inc.) under a light intensity of 1,000 μmol photons m^-2^ s^-1^.

### *In situ* H_2_O_2_ and O_2_^-^ detection

The *in situ* detection of H_2_O_2_ and O_2_^-^ was performed as previously described, with minor modification [72]. H_2_O_2_ in the roots was detected with 1 mg ml^-1^ of diaminobenzidine (DAB), while O_2_^-^ was measured using 10^-2^ M nitro-blue tetrazolium (NBT). For analysis, three roots from each sample were vacuum-infiltrated in 10 ml of solution for 2 h, after which they were cleared in boiling ethanol (95%) for 10 min. The samples were then stored and examined in 95% ethanol.

### MDA and proline content measurement

The MDA content was determined as previously described [47]. Briefly, approximately 0.5 g of fresh leaves were homogenized in 10 ml of 10% trichloroacetic acid (TCA) and then centrifuged at 12,000 ×g for 10 min. Next, 2 ml of 0.6% thiobarbituric acid in 10% TCA were added to 2 ml of the supernatant. The mixture was subsequently heated in boiling water for 30 min, then quickly cooled in an ice bath. After centrifugation at 10,000 ×g for 10 min, the absorbance of the supernatant at 450, 532, and 600 nm was determined. The MDA concentration was reported as nmol g^-1^ fresh weight (FW).

Proline content was measured as previously reported [73], with slight modification. Briefly, approximately 0.2 g of fresh leaves was homogenized in 10 ml of 3% aqueous sulphosalicylic acid, after which the homogenate was centrifuged at 2,000 ×g for 10 min. Next, 2 ml of the extract was incubated with 2 ml of acidic-ninhydrine and 2 ml of glacial acetic acid for 1 h in boiling water, after which the reaction was terminated in an ice bath. The reaction mixture was then extracted with 4 ml toluene and mixed vigorously with a test tube stirrer for 15–20 s. The chromophore containing toluene was subsequently aspirated from the aqueous phase and warmed to room temperature, after which the absorbance was read at 520 nm using toluene as a blank. The proline concentration was determined from a standard curve and calculated as μg g^-1^ FW.

### Antioxidant enzyme activity determination

Antioxidant enzymes were extracted using a previously described method [48]. The activities of CAT, APX, GR, and SOD were measured spectrophotometrically by monitoring the change of absorbance at 240, 290, 340 and 560 nm, respectively [74,75].

### Total protein extraction

Total proteins were extracted from *E*. *cerassipes* using a previously described method [76], with slight modification. Briefly, approximately 1 g powder of fresh leaves was homogenized with 5 mL TRIzol at 25°C for 5 min. Next, 1 mL chloroform was added and the mixtures were allowed to stand at −20°C for 5 min. Following centrifugation at 4°C and 12,000 ×g for 10 min, the supernatants were removed and the lower phases were mixed with isometric isopropanol and allowed to stand at −20°C for 2 h. The mixtures were then centrifuged at 4°C and 12,000 ×g for 10 min, after which the supernatants were removed. Next, the precipitates were washed three times with isopropanol and dried at 25°C, after which they were dissolved in denaturation buffer (7 M urea, 2 M thiourea, 4% (w/v) 3-[(3-cholamidopropyl)-dimethylammonio]-1-propane sulfonate, and 60 mM DTT) for 1 h with intermittent shaking.

### Protein 2-DE

Protein 2-DE was performed as previously described [47]. A total of 1,200 μg of proteins extracted from each sample were first separated by isoelectric focusing (IEF) using gel strips with a pH gradient of 4 to 7 (Immobiline Dry Strip, pH 4–7 NL, 17 cm; BioRad, Hercules, CA, USA). The strips were rehydrated for 14 h in 320 ml of dehydration buffer and then focused at 20°C for a total of 64 kV-h with a PROTEAN IEF Cell system (Bio-Rad). After IEF, the strips were equilibrated for 20 min, first in equilibration buffer I [6 M urea, 0.375 M Tris (pH 8.8), 2% (w/v) SDS, 20% (v/v) glycerol, and 2% (w/v) DTT) and then in equilibration buffer II [6 M urea, 0.375 M Tris (pH 8.8), 2% (w/v) SDS, 20% (v/v) glycerol, and 2% (w/v) iodoacetamide]. The equilibrated strips were then placed over 12.5% (w/v) sodium dodecyl sulfate-polyacrylamide gel electrophoresis (SDS-PAGE) gels for 2-DE at 25 mA for 5 h. The 2-De gels were stained with colloidal CBB.

### Spot digestion and protein identification for mass spectrometry analyses

Protein spot digestion and protein identification were performed as previously described [49]. Protein spots displaying significant changes in abundance following plant exposure to Cd stress were excised manually from colloidal CBB-stained 2-DE gels using sterile pipette tips. Spots were transferred to 1.5-ml sterile tubes, destained with 50 mM NH_4_HCO_3_ for 1 h at 40°C, reduced with 10 mM DTT in 100 mM NH_4_HCO_3_ for 1 h at 60°C, and incubated with 40 mM iodoacetamide in 100 mM NH_4_HCO_3_ for 30 min. Gels were then minced, air-dried, and rehydrated in 12.5 ng μl^-1^ sequencing-grade modified trypsin (Promega, Fitchburg, WI, USA) in 25 mM NH_4_HCO_3_ overnight at 37°C. Tryptic peptides were extracted three times from the gel grains using 0.1% trifluoroacetic acid (TFA) in 50% acetonitrile. Supernatants were concentrated to approximately 10 ml using a SpeedVac (Thermo Fisher, Waltham, MA, USA) and then desalted using reversed-phase ZipTip pipette tips (C18, P10; Millipore, Billerica, MA, USA). Peptides were eluted with 50% acetonitrile and 0.1% TFA.

Lyophilized peptide samples were dissolved in 0.1% TFA, and MS analysis was conducted using a 4800 Plus MALDI-TOF/TOF Proteomics Analyzer (Applied Biosystems, Foster City, CA, USA). MS acquisition and processing parameters were set to reflector-positive mode and an 800–3,500-Da acquisition mass range, respectively. The laser frequency was 50 Hz, and each sample spectrum was acquired over 700 laser pulses. For secondary MS analysis, four to six ion peaks with signal-to-noise ratios exceeding 100 were selected from each sample as precursors. TOF/TOF signal data for each precursor were then accumulated from 2,000 laser pulses. Primary and secondary mass spectra were transferred to Excel files and compared against a non-redundant NCBI protein database restricted to Viridiplantae (i.e., green plants) using the MASCOT search engine (www.matrixscience.com). The following search parameters were used: no molecular weight restriction, one missed trypsin cleavage allowed, iodoacetamide-treated cysteine, oxidation of methionine, a peptide tolerance of 100 ppm, and an MS/MS tolerance of 0.25 Da. Protein identifications were validated manually based on at least three matching peptides. Keratin contamination was removed, and the MOWSE threshold was set above 40 (*P*<0.05). Only significant hits in the MASCOT probability analysis were accepted as protein identifications.

### Expression analysis and functional classification

After staining with Coomassie Brilliant Blue, the 2-DE gels were scanned and the images were used to analyse the proteins expression using the PDQuest 2D analysis software (BioRad) based on their relative volumes. The volume of each spot was normalized [77] to compensate for subtle differences in sample loading or gel staining/destaining during individual experiments. Proteins with expressions that varied by at least 1.5-fold were regarded as differentially expressed. Functional classification of differentially expressed proteins was conducted according to Blast2Go [78].

### Statistical analysis

Statistical analyses were performed using SPSS version 18.0. ANOVA was used to identify differences among treatments (Tukey’s test, *P*<0.05).

## Supporting Information

S1 Fig The first set of 2-DE analysis of four *E*. *crassipes* samples treated with 100 mg/L Cd for different times. (TIF)

S2 Fig The second set of 2-DE analysis of four *E*. *crassipes* samples treated with 100 mg/L Cd for different times. (TIF)

S3 Fig The third set of 2-DE analysis of four *E*. *crassipes* samples treated with 100 mg/L Cd for different times. (TIF)
