# Supplementary figures and images for: Physiological and Proteomics Analyses Reveal the Mechanism of Eichhornia crassipes Tolerance to High-Concentration Cadmium Stress Compared with Pistia stratiotes
Source: PLoS One. 2015 Apr 17;10(4):e0124304. doi: 10.1371/journal.pone.0124304 (PMC4401520; doi:10.1371/journal.pone.0124304)

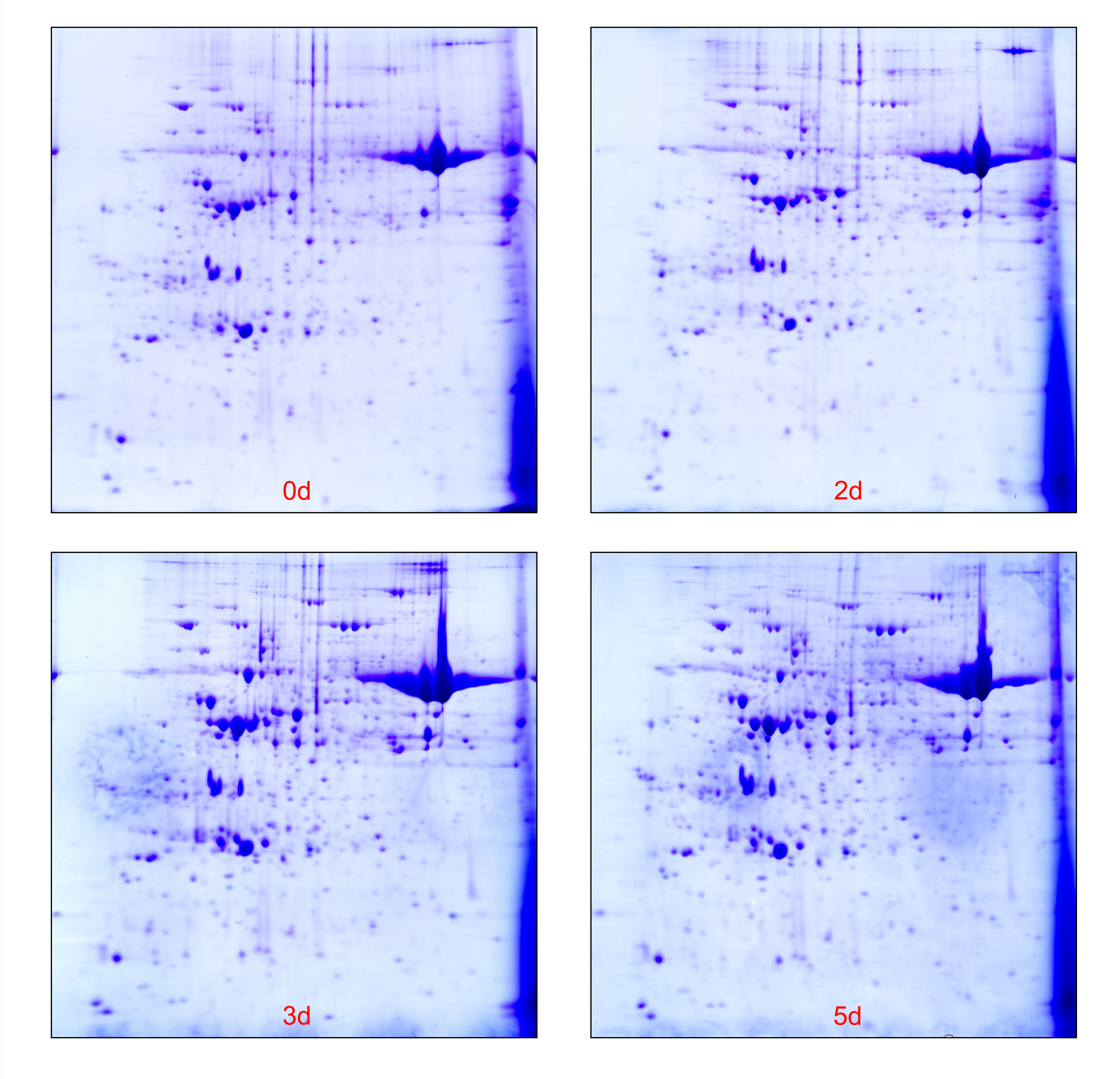

Supplement: S1 Fig — (TIF) [file pone.0124304.s001.tif]

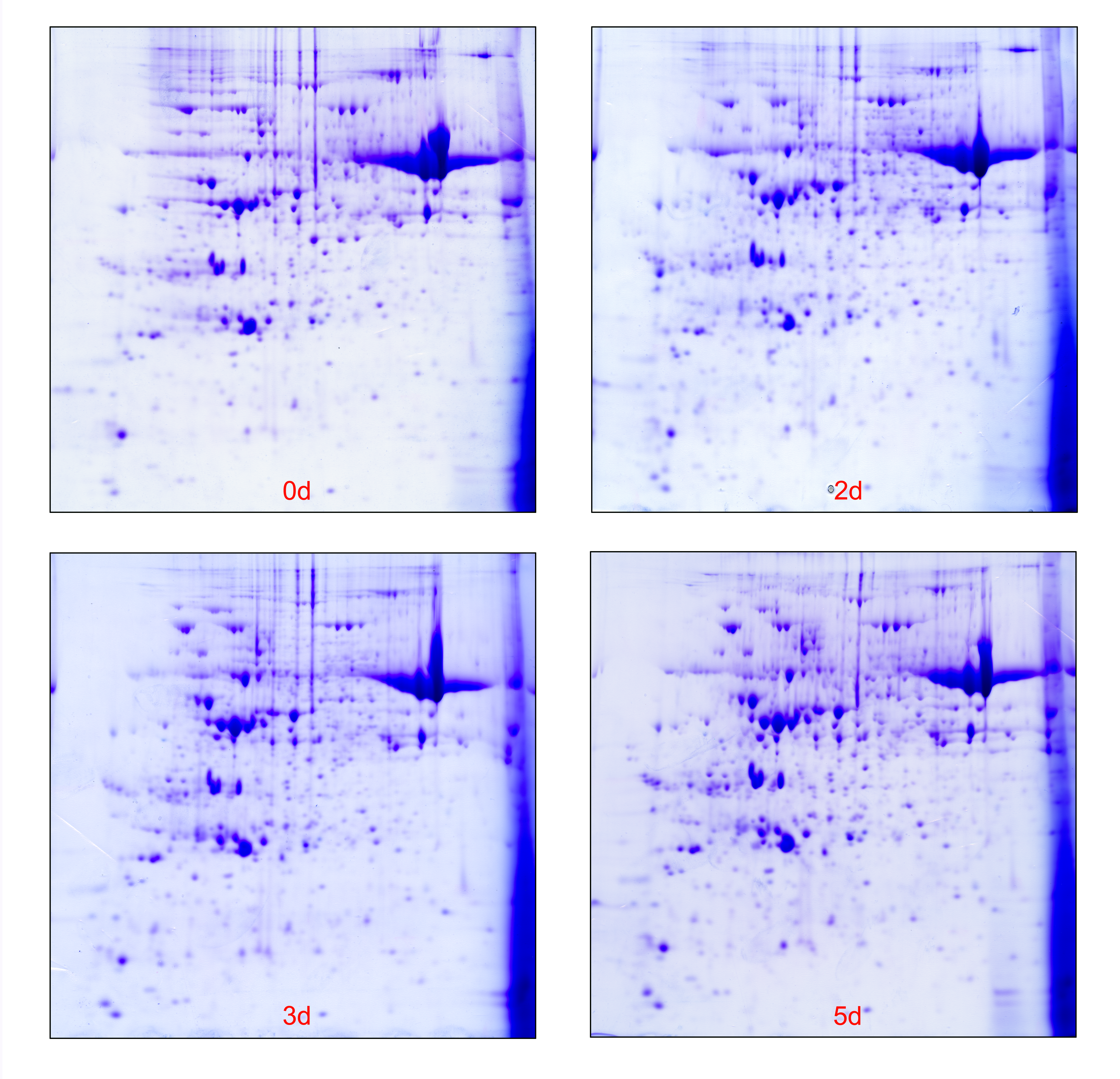

Supplement: S2 Fig — (TIF) [file pone.0124304.s002.tif]

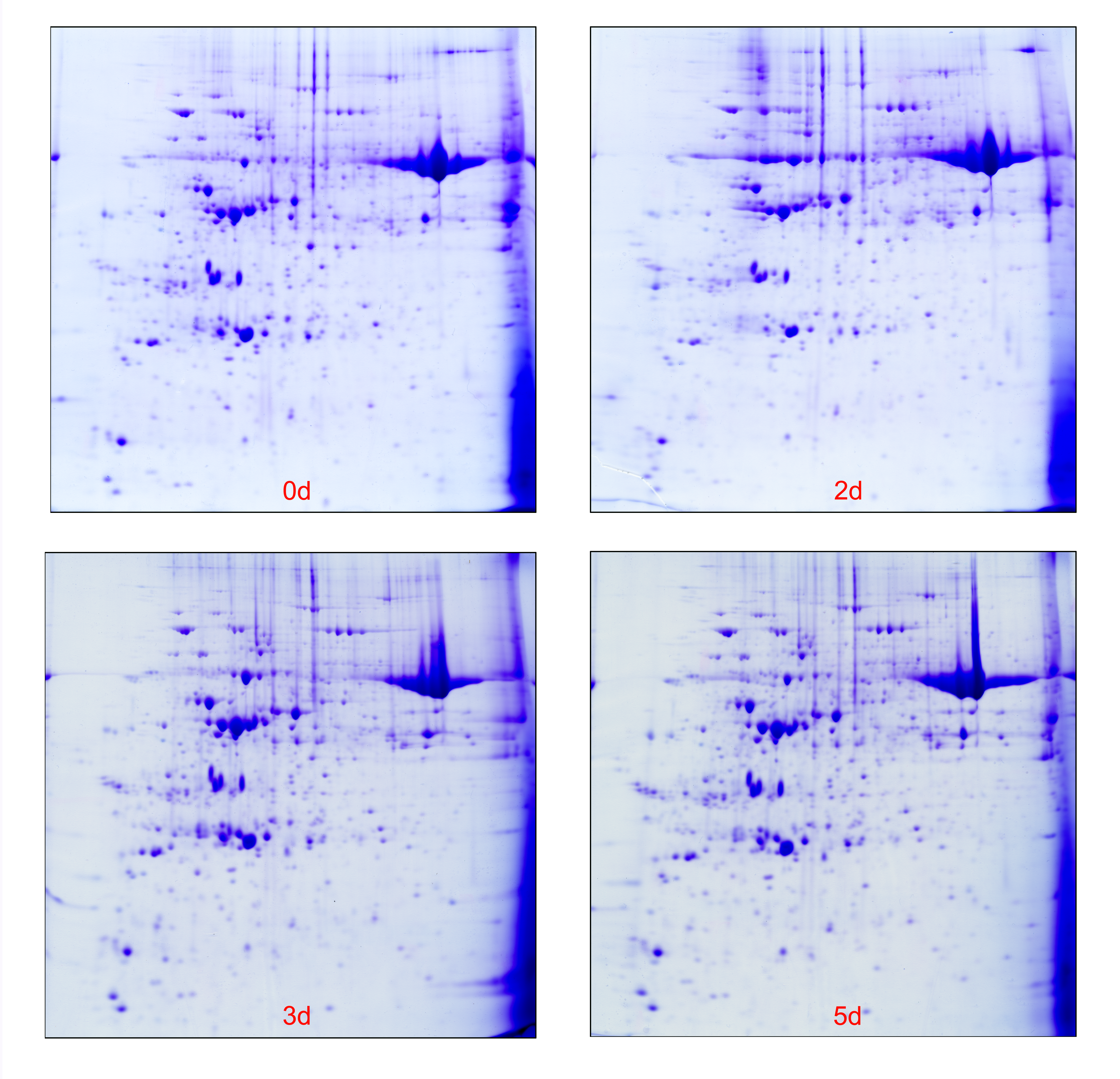

Supplement: S3 Fig — (TIF) [file pone.0124304.s003.tif]
